# ECONSULT IMPROVES ACCESS TO SPECIALIST ADVICE FOR PRIMARY CARE PROVIDERS

**DOI:** 10.1093/geroni/igac059.255

**Published:** 2022-12-20

**Authors:** Celeste Fung, Clare Liddy

**Affiliations:** University of Ottawa, Department of Family Medicine, Ottawa, Ontario, Canada; University of Ottawa, Ontario, Ontario, Canada

## Abstract

Dr. Liddy will present an overview of the Champlain BASE™ eConsult service, with a focus on its implementation in long-term care (LTC). eConsult is a secure web-based tool that enables providers to communicate electronically with specialists concerning a patient’s care. Evaluation of over 100,000 cases has consistently demonstrated positive impacts on access to care: PCPs receive responses in a median of 0.9 days, and two-thirds of cases are resolved without the patient needing a face-to-face specialist visit. A cross-sectional analysis of eConsult cases sent from LTC settings in 2018 demonstrated similar response times and referral avoidance to the larger sample (a median response time of 0.6 versus 0.9 days; proportion of cases resolved without a specialist visit 70% versus 67%). These findings, coupled with eConsult’s proven success at improving access to and equity of care, demonstrate the importance of supporting eConsults expansion.

